# 5-alpha-reductase inhibitor therapy postpones urine retention and prostate surgery in patients with prostate enlargement and a maximum uroflow rate of less than 15 ml/sec

**DOI:** 10.1371/journal.pone.0175356

**Published:** 2017-04-10

**Authors:** Wenhsu Lin, Shangsen Lee, Jengyuan Wu, Yuhung Kuo, Tengfu Hsieh

**Affiliations:** 1 Department of Urology, Nantou Hospital, Ministry of Health and Welfare, Nantou, Taiwan; 2 Department of Urology, Taichung Tzu Chi Hospital, Buddhist Tzu Chi Medical Foundation, Taichung, Taiwan; 3 Department of Surgery, Taichung Tzu Chi Hospital, Buddhist Tzu Chi Medical Foundation, Taichung, Taiwan; 4 School of Medicine, Tzu Chi University, Hualian, Taiwan; 5 Department of Research, Taichung Tzu Chi Hospital, Buddhist Tzu Chi Medical Foundation, Taichung, Taiwan; Cedars-Sinai Medical Center, UNITED STATES

## Abstract

**Background:**

This study investigated the risk of transurethral resection of prostate (TURP) and acute urine retention (AUR) in relation to 5-alpha-reductase inhibitor (5ARI) therapy.

**Methods:**

We identified 22,687 patients who were newly diagnosed with PE and low urinary tract symptoms (LUTS) between January 1, 2002 and December 31, 2011. We further classified study subjects who had moderate to severe LUTS and a maximum uroflow rate of less than 15ml/sec into three groups by their defined daily dose (DDD) of 5ARI used. The control group consisted of 7–28 cumulative DDD (cDDD) 5ARI users, while the short-term treatment group was 29-179cDDD 5ARI users, and the long-term treatment group was users of more than 180cDDD 5ARI. Each patient was monitored to identify those who subsequently developed TURP and AUR.

**Results:**

TURP and AUR are detected in 5.6% of control group, 7.6% of short-term treatment group and 5.5% of long-term treatment group during 10-year follow up. Compared with the control group, there was no difference in the risk of TURP and AUR in the short-term and long-term treatment groups (HR = 1.41, 95% CI 0.76 to 2.62 and HR = 0.81, 95% CI 0.42 to 1.56, respectively).

**Conclusion:**

5ARI therapy did not change the risk of TURP and AUR events in patients with PE, moderate to severe LUTS and a maximum uroflow rate of less than 15 ml/sec in 10 years of follow-up. But long-term 5ARI used can postpone AUR and TURP for 8.16 months.

## Introduction

5-alpha-reductase inhibitor therapy (5ARI) is a standard treatment for prostate enlargement (PE) with lower urinary tract symptoms (LUTS) [1–3]. Because of its effectiveness in decreasing prostate size, the prevalence of 5ARI use for PE with LUTS has steadily increased [4]. Traditionally, LUTS have been related to bladder outlet obstruction as a result of PE. But recent studies have shown, however, that LUTS are not necessarily related to pathologies of the prostate [5]. Moreover, the causes of LUTS are multifactorial [5]. Age, bladder function and underlying chronic medical condition are also playing important role in LUTS. Once the LUTS deteriorating, the effectiveness of 5ARI is unclear.

Furthermore, our previous study evaluated the adverse effect of 5ARI therapy in Taiwan, finding that clinicians tended to use 5ARI treatment for older PE patients and those with a higher Charlson Comorbidity Index Score (CCIS) [6]. This population differs from previous reports of the results of 5ARI treatment [7–9], and the effectiveness of 5ARI therapy in this population is still not clear. In the other hand, our previous study indicated that maximum urine flow rate of less than 15ml/sec is a risk factor of urinary retention and subsequent prostate surgery in BPH patients receiving alpha-1 blocker therapy [10]. However, it is not clear whether 5ARI treatment can decrease the risk of urinary retention and subsequent prostate surgery in these patients in our previous study.

Taiwan implemented a National Health Insurance (NHI) program in 1995. Enrollment in this government-run, universal, single-payer insurance system is mandatory, and currently up to 99% of Taiwan’s 23 million residents receive medical care through the NHI program [11]. Taiwan’s NHI regulates treatment with 5-alpha-reductase inhibitors as a second line treatment for PE with LUTS [12]. As described in detail previously, the NHI’s 5ARI reimbursement criteria before 2013 were (1) moderate to severe signs and symptoms (IPSS >7) of bladder outlet obstruction (BOO) after alpha 1-adrenergic blockers treatment, a maximum urine flow rate of less than 15ml/sec or an enlarged prostate volume of more than 20 mL as measured by transrectal ultrasound, (2) excluding the possibility of prostate cancer, and (3) good response to the 5ARI (maximum urine flow rate increased or prostate volume decreased) in the first year of treatment [12]. Patients must meet all three criteria for 5ARI treatment. Under these reimbursement criteria, 5ARI therapy has been used for more than 10 years, but few studies have evaluated the long-term results of this therapy in Taiwan.

This study examines the prevention of urine retention and prostatectomy after 5ARI treatment in patients with a maximum uroflow rate below 15 ml/sec. A data set including ten years of records from Taiwan’s well-validated National Health Insurance Research Database (NHIRD) [13–16] is used to investigate the long-term outcomes of 5ARI therapy.

## Material and method

### Data source and ethics statements

Our study used data in the National Health Insurance Research Database (NHIRD) from 1 January, 2002 to 31 December, 2011. The NHIRD is provided by Taiwan’s National Health Research Institutes, and is made available to researchers who meet the criteria for access to confidential data (http://nhird.nhri.org.tw/date_01_en.html). The Institutional Review Board of Taichung Tzu Chi General Hospital in Taiwan approved the study protocol (REC103-43). Because the personal information of the individuals in this study had been scrambled cryptographically to ensure anonymity by Taiwan’s National Health Research Institutes, the review board waived the need for written consent.

### Study design

We used the International Classification of Diseases, 9th Revision, Clinical Modification (ICD-9-CM) diagnosis codes and ICD-9-CM treatment codes in this study. The defined daily dose (DDD) is a unit for measuring a prescribed amount of a drug; it is the assumed average daily maintenance dose of a drug consumed for its main indication in adults [17]. The cumulative DDD (cDDD), which indicates the duration of exposure, was estimated as the sum of dispensed DDD of 5ARI.

All patients with newly-diagnosed BPH (ICD-9-CM code 600.xx) and followed-up between 1 January, 2003 to 31 December, 2011 were included. Patients who received 5ARI inhibitor therapy, had moderate to severe LUTS (IPSS >7) and had a uroflowmetry study before 5ARI therapy were identified as the study cohort. The date of initiation of 5ARI therapy was used as the patient’s index date. The control group consists of 7-27cDDD 5ARI users, while the short-term treatment group is 28-180cDDD 5ARI users, and the long-term treatment group consists of patients using more than 180cDDD of 5ARI. According to Taiwan’s NHI regulates treatment with 5ARI as described in detail previously [12], these patients with 5ARI medication had moderate to severe signs and symptoms (IPSS >7) of bladder outlet obstruction (BOO) after alpha 1-adrenergic blockers treatment and a maximum urine flow rate of less than 15ml/sec. As our previous study definition, the alpha 1-adrenergic blockers user was defined as alpha 1-adrenergic blockers exposure more than 7cDDD [10]. The alpha 1-adrenergic blockers were classified into non-selective alpha 1-adrenergic blockers (terazosin, doxazosin, afluzosin, phenoxybenzamine) and selective alpha 1-adrenergic blockers (tamsulosin) [10].

The study excluded patients with newly-diagnosed acute urine retention (n = 41), who had received a transurethral resection of prostate (TURP) or had acute urine retention (AUR) before the index date, or 6 months of the last date of 5ARI medication in short-term treatment group and control group, or 6 months of 5ARI medication in long-term treatment group (n = 200), who had a follow-up duration of less than 6 months (n = 211) or less than 7cDDD (n = 41) of 5ARI. We further classified the study subjects into three groups by their DDD of 5ARI used. Data in S1 Fig displays a flowchart diagram explaining the numbers of individuals at each stage of the study.

Independent variables were gender, co-morbid disorders, geographical area of residence, urbanization level, and socio-economic status (SES).

### Research outcomes

As described in detail previously, the main outcome of the study was the occurrence of TURP (ICD-9-CM Code: 60.21 and 60.29), which was determined by linking records with inpatient care data in the NHIRD or acute urine retention, which was determined by linking records with inpatient care data or out-patient service claims of urine retention diagnosis (ICD-9-CM Code: 788.2) and urethral catheterization (47013C, 47014C) [10, 18]. The event is defined after 6 months of the last date of 5ARI medication in short-term treatment group and control group or after 6 months of 5ARI medication in long-term treatment group because 80% of AUR cases tended to recur within 6 months [19].

### Other variables

Because the government-stipulated minimum wage for full-time employees in Taiwan since 2006 was US$528, we set the low income as the equivalent of US$528. We further classified our study subjects into three groups according to income: (1) low SES (income less than US$528 per month); (2) moderate SES (income between US$528 to 833 per month); and (3) high SES (income more than US$833 per month) [20]. We classified geographic region of residence as northern, central, southern, and eastern Taiwan.

The levels of urbanization in our study had been described in other study [21]. Briefly, we classified the regions where the study subjects resided in Taiwan into 7 levels of urbanization. The urbanization level was categorized as urban (urbanization level: 1), suburban (urbanization level: 2–4) and rural (urbanization level: 5–7).

### Statistical analysis

All data analysis was performed by using commercial statistical software (SPSS version 15, SPSS Inc., Chicago, IL, USA). Pearson’s chi-square test was used for categorical variables such as gender, SES, geographic region of residence, and co-morbidities. One-way analysis of variance (ANOVA) was used for continuous variables analyzing. Kaplan-Meier survival curve was used to estimate the cumulative risk of TURP or urine retention.

We used a time-dependent Cox proportional hazards regression model adjusted for patient characteristics to calculate the hazard ratios (HRs) and 95% confidence intervals (CIs) of 5ARI use with subsequent TURP or urine retention. Two-tailed P values<0.05 were considered statistically significant.

## Results

The clinical and demographic characteristics of the study subjects were shown in Table 1. We had 624 patients in the control group, 1923 in the short-term treatment group and 1586 in the long-term treatment group. The three groups were consistent in terms of demographic characteristics and selected morbidities except age. Alpha-1 adrenergic blocker usage was very common in three groups: 57.7%, 68.7% and 76.4%, respectively in the control, short-term treatment and long-term treatment groups used non-selective alpha-1 adrenergic blockers, along with 60.6%, 70.8% and 73.3% using selective alpha-1 adrenergic blockers. Furthermore, crossover use of selective and non-selective alpha-1 adrenergic blocker was very common: 38.6%, 46.7% and 52.9%, respectively in the control, short-term treatment and long-term treatment groups.

**Table 1:** Demographic Characteristics (*n* = 4133).

Variables	Control, n (%)	Short-term treatment, n (%)	Long-term treatment, n (%)
**Patient No.**	624	1923	1586
**Mean age, years(±SD)**	64.6±16.0	68.3±10.7*	70.3±9.4*^†^
**CCIS score**			
0–1	456(73.1)	1377(71.6)	1120(70.6)
2–3	120(19.2)	421(21.9)	377(23.8)
> 3	48(7.7)	125(6.5)	89(5.6)
**Comorbidity**			
Diabetes	111(17.8)	317(16.5)	231(14.6)
Stroke	74(11.9)	220(11.4)	193(12.2)
Multiple sclerosis	1	0	0
Parkinsonism	11(1.8)	38(2.0)	29(1.8)
UTI	70(11.2)	309(16.1)	250(15.8)
**α1- adrenergic blockers used**			
Non-selective	360(57.7)	1322(68.7)	1212(76.4)
Selective	378(60.6)	1361(70.8)	1162(73.3)
Both	241(38.6)	898(46.7)	839(52.9)
**Socioeconomic status**			
Low	272(43.6)	695(36.1)	633(39.9)
Moderate	203(32.5)	610(31.7)	379(23.9)
High	149(23.9)	618(32.1)	574(36.2)
**Urbanization**			
Urban	197(31.6)	631(32.8)	601(37.9)
Suburban	270(43.3)	810(42.1)	687(43.3)
Rural	157(25.2)	482(25.1)	298(18.8)
**Geographic region**			
Northern/Central	459(73.6)	1363(70.9)	1127(71.1)
Southern/Eastern	165(26.4)	560(29.1)	459(28.9)

Chi-square test; One-way ANOVA. Control:5ARI exposure 7~27 cDDD; short-term treatment: 5ARI exposure 28~180cDDD, long-term treatment: 5ARI exposure >180cDDD

*Compared with control group, p-value<0.05

† Compared with short-term treatment, p-value<0.05

**Fig 1:**
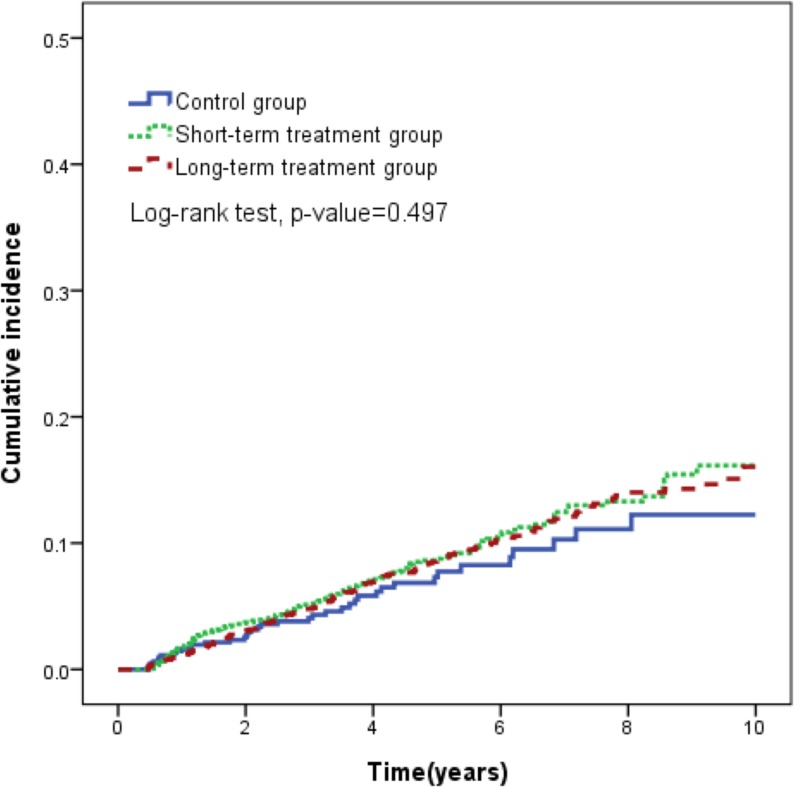
Cumulative incidence of TURP and AUR estimated by the Kaplan-Meier method for the control, short-term treatment, and long-term treatment groups. Abbreviations: 5ARI, 5-alpha reductase inhibitor; AUR, acute urine retention; cDDD, cumulative defined daily dose; TURP, transurethral resection of prostate

At the end of the follow-up period, 145 (3.5%) patients had TURP, including 16 (2.6%) in the control group, 64(3.3%) in the short-term treatment group and 65 (4.1%) in the long-term treatment group. Moreover, 197 (4.8%) patients had AUR, including 23 (3.7%) in the control group, 88(4.6%) in the short-term treatment group and 86 (5.4%) in the long-term treatment group (Table 2). As shown in the Kaplan-Meier curve in Fig 1, the 10-year risk of developing AUR and TURP was consistent for all three groups (log-rank test *p* = 0.497).

**Table 2:** The cumulative rate of TURP and AUR in three groups for 10 years.

Characteristics	Event (%)		
	TURP	AUR	TURP+AUR
Control group, n = 624	16(2.6)	23(3.7)	39(6.3)
Short-term treatment, n = 1923	64(3.3)	88(4.6)	152(7.9)
Long-term treatment, n-1586	65(4.1)	86(5.4)	151(9.5)
**Total, n = 4133**	145(3.5)	197(4.8)	342(8.3)

Control:5ARI exposure 7~27 cDDD; short-term treatment: 5ARI exposure 28~180cDDD, long-term treatment: 5ARI exposure >180cDDD. Abbreviations: 5ARI, 5-alpha reductase inhibitor; AUR, acute urine retention; TURP, transurethral resection of prostate

When adjusted for age and comorbidities, Multivariate Cox proportional hazard regression analysis revealed the three groups had the same risk of developing AUR and TURP (Table 3). The adjusted HR was 1.18 (95% CI: 0.83 to 1.69) for the short-term treatment group and 1.06 (95% CI: 0.74 to 1.52) for the long-term treatment group. Moreover, we found that age (HR = 1.03, 95% CI: 1.02 to 1.04) is a significant factor for incidence of TURP and AUR.

**Table 3:** Cox model measured hazard ratio and 95% confidence intervals of TURP and AUR.

Characteristics	Crude		Adjusted	
	HR	95% CI	HR	95% CI
**5ARI dosage**				
Control	1	ref	1	ref
Short-term treatment	1.24	0.87 to 1.76	1.18	0.83 to 1.69
Long-term treatment	1.17	0.82 to 1.67	1.06	0.74 to 1.52
**Age**	1.03	1.02 to 1.05*	1.03	1.02 to 1.04*
**CCIS**	1.15	1.08 to 1.22*	1.11	1.03 to 1.20 ^†^
**Comorbidity**				
Diabetes	1.31	1.00 to 1.71#	1.09	0.81 to 1.46
Stroke	1.03	0.74 to 1.43	0.72	0.50 to 1.03
Parkinsonism	1.12	0.53 to 2.36	0.92	0.43 to 1.95
UTI	1.33	1.01 to 1.75#	1.26	0.96 to 1.66
**Socioeconomic status**				
Low	1	ref	1	ref
Moderate	0.83	0.65 to 1.08	0.85	0.65 to 1.12
High	0.67	0.52 to 0.87 ^†^	0.90	0.68 to 1.20
**Urbanization**				
Urban	1	ref	1	ref
Suburban	1.48	1.14 to 1.92 ^†^	1.54	1.18 to 2.01 ^†^
Rural	1.60	1.19 to 2.14 ^†^	1.78	1.28 to 2.50 ^†^
**Geographic region**				
Northern/Central	1	ref	1	ref
Southern/Eastern	0.99	0.78 to 1.25	0.80	0.62 to 1.02

Abbreviation: 5ARI, 5-alpha reductase inhibitor; AUR, acute urine retention; CCIS, Charlson Comorbidity Index score; CI, confidence interval; HR, hazard ratio; TURP, transurethral resection of prostate; UTI, urinary tract infection. Adjusted HR: adjusted for 5ARI dosage, age, CCIS, geographic region, socioeconomic status, and comorbidity of Diabetes, Stroke, Parkinsonism and UTI in Cox proportional hazards regression

# p<0.05

† p<0.01

* p<0.001

We further analysis all the patients developing AUR or TURP in Table 4 to exam whether 5ARI can delay events happening. Because the maximal prostate volume decreasing happened after 5ARI exposure more than 180 cDDD, we only classified patient into long-term treatment group or not. The data indicated that long-term 5ARI treatment can delay patients developing AUR or TURP in 8.16 months (SE = 2.93, p<0.01).

**Table 4:** Multiple linear regression of follow time in patients with event (n = 352).

Parameter	B	Standard error	P-value	95% CI	
Intercept	70.00	14.55	< .01	41.38	98.62
**5ARI dosage**					
Control and short-term treatment group	1.00				
Long-term treatment group	8.16	2.93	0.01	2.40	13.92

Abbreviation: 5ARI, 5-alpha reductase inhibitor. Control:5ARI exposure 7~27 cDDD; short-term treatment: 5ARI exposure 28~180cDDD, long-term treatment: 5ARI exposure >180cDDD

## Discussion

After adjusting for age, insurance premiums, residential area, CCIS, and comorbidities, this nationwide population-based 10-year follow up study found no difference in the incidence of TURP and AUR in different 5ARI users with maximum uroflow rates of less than 15ml/sec. However, long-term 5ARI used can postpone AUR and TURP for 8.16 months, compare to non-user or short-term users with maximum urine flow rate of less than 15ml/sec. Age is an important factor for TURP and AUR in patients with a maximum uroflow rate of less than 15ml/sec. The findings provide a reference for the clinical treatment of PE patients with LUTS.

Taiwan’s NHI program has strict regulations regarding reimbursement for 5ARI (http://www.nhi.gov.tw/information/BBS_Detail.aspx?menu=9&menu_id=545&bulletin_id=1924). Reimbursement is granted only to patients who fit the reimbursement criteria[12]. Under such regulations, patients should have moderate to severe LUTS, symptoms of BOO, have low risk of prostate cancer, have previous treatment for PE with LUTS and a maximum uroflow rate of less than 15 ml/sec. These regulations also result in a homogeneous study population.

Urine retention is the key issue for patients with BPH with LUTS. Although prostate size or, more specifically, prostate urethra resistance, is the important factor for urine retention, urinary bladder contractility and function also play a major role. 5ARI therapy is well documented to reduce prostate size and LUTS symptoms, but few reports have documented its effectiveness in reducing prostate urethra resistance, improving bladder contractility and providing long-term functional improvement [22]. Moreover, urine retention is not only a problem for the urinary tract, but is also a predictor of patient’s systemic medical conditions. Many studies have indicated that high urine retention is associated with increased mortality rate [23, 24]. These results suggest that urine retention is associated with poor systemic patient medical condition. Our previous studies found that physicians are more prone to prescribing 5ARI in older patients with co-morbidities, and we suggest that 5ARI therapy is only marginally effective in these fragile patients.

Previous studies have found 5ARI therapy to be effectiveness against PE with LUTS [7, 25]. But Lepor et al. found that 5ARI therapy was not effective in treating men with PE [26]. The results of the present study support this finding.

In our previous study we disclosed that maximum urine flow rate of less than 15ml/sec is a risk factor of urinary retention and subsequent prostate surgery in BPH patients receiving alpha-1 blocker therapy. However, the results of present study indicated that, in patients with maximum urine flow rate of less than 15ml/sec, the majority effect of long-term 5ARI therapy is to postpone AUR and TURP for 8.16 months and 5ARI treatment can’t change the incident of AUR and TURP in 10 years of follow-up.

Some limitations to the present study should be noted. First, we were unable to obtain the actual uroflowmetry pattern for all study subjects. We only enrolled the patients with maximum uroflow rate of less than 15ml/sec into study. All patients underwent continuous 5ARI therapy, and the maximum uroflow rate was monitored every 6 months as NHI regulations require improvement in the maximum uroflow rate during the first year of 5ARI treatment.

Second, some patients who failed to show improved maximum uroflow in the first year of treatment, and were thus excluded from further NHI-covered treatment, may have paid for continued treatment out of pocket, and thus may have been inappropriately classified into the short-term treatment group. On the other hand, members of the long-term treatment group may exhibit poor compliance with the prescribed course of treatment. However, the control group membership was accurate because 5ARI prescription is strictly regulated by the NHI, and only patients fitting the specific criteria are eligible to receive reimbursement.

Third, the study lacks information related to the severity of LUTS, such as prostate size, serum prostate specific antigen level, urinary bladder derusor contractility and function. Further studies linking administrative data and primary hospitalization information are warranted. However, given the magnitude and statistical significance of the observed effects in this study, these limitations are unlikely to compromise the results.

Fourth, the study does not determine what kind of patients benefit from 5ARI. Early use of 5ARI may produce improved AUR prevention results, or 5ARI treatment may be more effective in patients with fewer comorbidities, but this cannot be evaluated given the retrospective study design.

## Conclusions

The results of the present study show that 5ARI therapy did not decrease the risk of TURP and AUR events in patients with PE and a maximum uroflow rate of less than 15 ml/sec in 10 years of follow-up. The majority effect of long-term 5ARI therapy is to postpone the AUR or TURP for 8.16 months. This is the real world data in Taiwan although it may be different from other clinical trials. Further mechanistic research is needed.

## Supporting information

S1 Fig Recruitment process for subjects with 5-alpha-reductase inhibitor therapy from 1 million random samples in the National Health Insurance Research Database (NHIRD). (DOCX)
