# Supplementary material for: 5-alpha-reductase inhibitor therapy postpones urine retention and prostate surgery in patients with prostate enlargement and a maximum uroflow rate of less than 15 ml/sec
Source: PLoS One. 2017 Apr 10;12(4):e0175356. doi: 10.1371/journal.pone.0175356 (PMC5386275; doi:10.1371/journal.pone.0175356)
Supplement: S1 Fig — (DOCX) [file pone.0175356.s001.docx]

S1 Fig. Recruitment process for subjects with 5-alpha-reductase inhibitor therapy from 1 million random samples in the National Health Insurance Research Database (NHIRD)

Abbreviations: 5ARI, 5-alpha reductase inhibitor; AUR, acute urine retention; cDDD, cumulative defined daily dose; TURP, transurethral resection of prostate

Exclude:

1. TURP occurred before index day + 180 days or in latency period*, n=41
2. AUR occurred before index day + 180 days or in latency period*, n=159
3. Follow up less than 6 months, n=211
4. 5ARI used less than 7 cDDD, n=41

* latency period: 6 months of the last date of 5ARI medication in short-term treatment group and control group or initially 6 months of 5ARI medication in long-term treatment group.

Uroflowmetry study before 5ARI used, n=1858

Newly-diagnosed of BPH patients between 1 January, 2002 and 31 December, 2011 from one million random sample of NHIRD, n=22687

TURP/AUR or not

Short-term treatment group (29-179cDDD 5ARI user), n=628

Long-term treatment group (≥180cDDD 5ARI user), n=545

Control group (7-28cDDD 5ARI user), n=233
